# Optical Coherence Tomography Angiography of the Choriocapillaris in Age-Related Macular Degeneration

**DOI:** 10.3390/jcm10040751

**Published:** 2021-02-13

**Authors:** Jackson Scharf, Giulia Corradetti, Federico Corvi, SriniVas Sadda, David Sarraf

**Affiliations:** 1 Vagelos College of Physicians and Surgeons, Columbia University, New York, NY 10032, USA; jms2455@cumc.columbia.edu; 2 Department of Ophthalmology, Stein Eye Institute, University of California, Los Angeles, CA 90095, USA; gcorradetti@mednet.ucla.edu (G.C.); ssadda@doheny.org (S.S.); 3 Doheny Eye Institute, University of California, Los Angeles, CA 90033, USA; federico.corvi@yahoo.it; 4 Eye Clinic, Department of Biomedical and Clinical Science “Luigi Sacco”, Sacco Hospital, University of Milan, 20157 Milano, Italy; 5 Department of Ophthalmology, Greater Los Angeles VA Healthcare Center, Los Angeles, CA 90073, USA

**Keywords:** age-related macular degeneration, optical coherence tomography angiography, choriocapillaris, OCT-A, retinal imaging, macular neovascularization, choriocapillaris quantification, flow deficit

## Abstract

The advent of optical coherence tomography angiography (OCTA) has allowed for remarkable advancements in our understanding of the role of the choriocapillaris in age-related macular degeneration (AMD). As a relatively new imaging modality, techniques to analyze and quantify choriocapillaris images are still evolving. Quantification of the choriocapillaris requires careful consideration of many factors, including the type of OCTA device, segmentation of the choriocapillaris slab, image processing techniques, and thresholding method. OCTA imaging shows that the choriocapillaris is impaired in intermediate non-neovascular AMD, and the severity of impairment may predict the advancement of disease. In advanced atrophic AMD, the choriocapillaris is severely impaired underneath the area of geographic atrophy, and the level of impairment surrounding the lesion predicts the rate of atrophy enlargement. Macular neovascularization can be readily identified and classified using OCTA, but it is still unclear if neovascularization features with OCTA can predict the lesion’s level of activity. The choriocapillaris surrounding macular neovascularization is impaired while the more peripheral choriocapillaris is spared, implying that choriocapillaris disruption may drive neovascularization growth. With continued innovation in OCTA image acquisition and analysis methods, advancement in clinical applications and pathophysiologic discoveries in AMD are set to follow.

## 1. Introduction

Innovation in ophthalmic imaging has led to remarkable advancements in our understanding of age-related macular degeneration (AMD). The relatively thin and highly vascularized choriocapillaris, critical to the pathophysiology of AMD, is located immediately posterior to the Bruch’s/retinal pigment epithelium complex and has historically been challenging to visualize with conventional imaging. With the advent of optical coherence tomography angiography (OCTA), however, blood flow in the choriocapillaris can be identified with far greater detail than ever before. This advancement has heralded critical new insights into the pathophysiology of both non-neovascular and neovascular AMD and is poised to become an important part of the clinical care of AMD patients.

OCTA is a relatively new imaging modality that facilitates visualization of the retinal and inner choroidal circulation without the need for dye injection [1]. It employs motion contrast to detect blood flow and acquires three-dimensional volumetric information of the retina and choroid to provide high-resolution, depth-resolved segmentation of the different vascular layers, including the choriocapillaris [1].

This review discusses the basic principles of OCTA and defines the current role and relevance of OCTA-based choriocapillaris imaging in the assessment of eyes with both non-neovascular and neovascular AMD.

## 2. OCTA Analysis of the Choriocapillaris

### 2.1. Basic Principles of OCTA

OCTA is a noninvasive tool that creates a reconstruction of the retinal capillary and inner choroidal vasculature [2,3], by recognizing the intrinsic movement of particles in these tissues. The device captures a dense volume of OCT scans at the same location and then detects differences between the scans over a short, designated time interval. A calculation is performed for each pixel in every frame to identify which pixels are changing (in phase and/or amplitude) over time, thereby isolating or contrasting moving structures. OCTA images combine the structural information of a standard OCT scan with blood flow visualization. The moving elements are commonly coded as bright/white pixels on the OCTA scans to represent blood flow, while the dark areas represent areas with blood flow below the decorrelation threshold referred to as “flow deficits” [4,5,6].

Different devices employ unique calculation methods to analyze OCT intensity information. Algorithms used by OCTA systems can be divided into three categories: (A) angiography based on both the phase and amplitude components of the OCT signal (OMAG or OCT microangiography-complex; CODAA, complex OCT signal difference analysis angiography), (B) angiography based on only the amplitude of the OCT signal (SSADA, split spectrum amplitude decorrelation algorithm; OCTARA, OCTA ratio analysis), and (C) angiography based on only the phase of the OCT signal (Doppler OCT) [7].

### 2.2. Spectral Domain OCTA versus Swept Source OCTA

Several OCTA systems are available in clinical practice and can be broadly divided into spectral domain (SD) and swept source (SS) devices. This distinction is based primarily on the wavelength of the device’s light source: 840 nm for SD and 1050 nm for SS devices. The difference in wavelength results in distinctive penetration of the signal through the RPE, drusen, and other blocking structures, thereby producing different visualizations of the choroidal layers [8]. SS-OCTA, with its longer wavelength, facilitates deeper penetration of the signal through the RPE, and through pathological structures such as drusen that shadow, providing better visualization of the choroid and a more detailed, high-resolution image [7]. Due to the relative limitations of the SD-OCTA system when compared to SS-OCTA, some researchers have excluded regions below drusen from quantitative analyses of the choriocapillaris when using SD-OCTA [9].

The differences in SS and SD OCTA, however, extend beyond their wavelengths. SD-OCTA is characterized by a broad bandwidth light source which is coupled with a spectrometer, while SS-OCTA is equipped with photodetectors and a tunable laser light source that operates through a range of frequencies. Furthermore, SS-OCTA is characterized by a faster rate of acquisition of the images, at 100,000–400,000 A-scans per second versus around 70,000 to 100,000 A-scans per second for most commonly available SD-OCTA systems. Considering that OCTA relies on decorrelation between sequentially acquired OCT B-scans, increased speed of acquisition allows for improved image quality. Both increased imaging speed and deeper penetration with SS-OCTA significantly improve the visualization of the choriocapillaris, which is particularly important in the case of AMD.

OCTA images can be altered by artifacts that complicate the accurate interpretation and quantification of the choriocapillaris [10]. To minimize motion artifacts while acquiring images, OCTA devices employ an active “Eye-Tracking” system. Artifacts can also be generated by the presence of structures casting shadows, such as drusen in eyes with AMD. Drusen can alternatively be associated with projection and *Z*-axis micro-motion artifacts, due to their highly reflective surface, generating a false positive flow signal termed “pseudoflow” [11], which can be mistaken for pathological neovascularization. Artifacts like these may also interfere with quantitative analysis of the choriocapillaris by generating a false positive flow deficit at the level of the OCTA choriocapillaris slab [12]. In order to compensate for projection artifacts, OCTA devices are equipped with projection artifact removal software. Media opacities pose another potential complication to OCTA image quality. These factors each play a particularly critical role in the quantification of choriocapillaris flow deficits, because poor quality images generate falsely diminished flow due to a reduced signal rather than true pathology, and are discussed in more detail below.

### 2.3. Quantification of the Choriocapillaris

Reliable quantification of the choriocapillaris has many complex challenges which have inspired numerous unique approaches. Imaging the choriocapillaris with OCTA is not as simple as positioning a slab in the proper anatomic location (between the Bruch’s membrane and the inner border of Sattler’s layer [13]). Several factors may influence this analysis [14]. In regard to segmentation, the outer border of the choriocapillaris may not be located at a consistent offset from Bruch’s membrane, which may introduce inaccuracy despite an otherwise “correct” anatomic segmentation. The thickness of the choriocapillaris is variable, measuring 10 μm under the fovea and 7 μm moving to the periphery with undulations [15]. Finally, differences may exist between devices. Quantitative OCTA metrics between instruments and scan patterns are not interchangeable, and several devices use the RPE band as the offset, resulting in increased variability related to fluctuations in the thickness of the RPE [16,17].

Considering these many factors, no consensus has been reached about the optimal position and thickness of the choriocapillaris slab, and diverse choriocapillaris segmentation strategies have been adopted. This variability has a profound impact on the consistency of studies in the literature, as quantitative choriocapillaris measurements may be significantly influenced by small differences in slab selection [18]. Byon et al. [19] found that the use of a Max projection with a slab positioned 21–31 μm below the RPE band centerline produced the most repeatable flow deficit measurements in normal eyes. More superficial slabs showed a hypointense region caused by inadvertent inclusion of the RPE band in the slab, while deeper slabs showed inadvertent inclusion of the choroidal stroma. These artifacts could theoretically be further accentuated if signal compensation strategies were applied as described below [20].

Image compensation is a recently proposed method to correct regions of signal loss using an inverted version of the corresponding en face structural OCT slab [2]. Images with signal compensation show fewer choriocapillaris flow voids and improved repeatability of measurement. This is particularly helpful in eyes with drusen to compensate for their masking effects [2]. It has recently been demonstrated, however, that this technique may alter the appearance of the thresholded images, creating the appearance of new flow deficits and causing others to disappear [20].

In addition to image compensation, averaging of multiple en face angiographic images after registration improves visualization of the choriocapillaris [21]. This technique improves image quality by reducing noise that could be misinterpreted as flow and annealing discontinuous vessel segments, thus improving visualization of the choriocapillaris and resolution of the intervascular spaces [12]. In particular, averaging has been shown to increase the measured vessel area density and decrease the number of flow voids, total flow void area, and average flow void size [22]. It should be considered, however, that this technique requires multiple scans with extra time and considerable patient cooperation.

Despite the recent progress in choriocapillaris image processing, OCTA images are subject to a number of common artifacts that can impact their interpretation. Eyes with choroidal disease are commonly affected by choriocapillaris segmentation errors that result in segmentation artifacts [23]. In these cases, semi-automated approaches with manual correction of the segmentation errors are commonly adopted. Quantitative analysis of the choriocapillaris can also be significantly affected by projection artifacts from the superficial retinal vessels. To mitigate these changes, several devices contain projection removal functions which remove the flow signal cast by overlying retinal vessels from the choriocapillaris slab. Many image processing strategies independent of the OCTA devices have also been adopted to eliminate these potentially confounding residual shadow or projection artifacts [9,24,25,26,27,28]. Motion artifacts created by body and eye movement and reduced signal strength of the images can also profoundly reduce the accuracy of quantitative analysis of the choriocapillaris [1,29]. Decreased image quality increases the frequency of artifacts and decreases repeatability of choriocapillaris flow deficit measurements. In consideration of these many variables, axial slab positions, reference offsets, projection artifact removal methods, signal strength and thresholding strategies must all be carefully considered when quantitatively analyzing the choriocapillaris [1,4,29,30,31,32].

Consideration of the anatomy of the choriocapillaris has led to discussion over which flow deficits are pathologic and which may be physiologic. Histologic studies show that the choriocapillaris has a different morphology in different regions of the retina: the submacular region has a dense honeycomb network of freely interconnected capillaries separated by septa, while the equatorial and peripheral regions have a polygonal lobular network [13,33,34]. The distance between capillaries also changes according to the region, ranging from 2 microns in the center to 20 microns in the periphery [13]. In consideration of this anatomy, some OCTA studies have suggested excluding flow deficits smaller than 24 µm in diameter when quantifying flow voids, as they may represent physiologic intercapillary gaps [2,3,4,8].

When quantifying the choriocapillaris, it is critical to create a threshold above which a pixel is considered to have blood flow, and below which a pixel is considered to have a flow deficit. Multiple thresholding methods have been proposed, each of which significantly impacts vessel density measurements [30,31]. One of the first proposed was Otsu’s global thresholding methodology, however it assumes a bimodal distribution of decorrelation in the image histogram, which may not be accurate when considering inner choroidal images [35,36]. Another approach is the mean outer retinal pixel value global threshold, which is based on the hypothesis that the outer retinal layer and inner choroidal layer manifest the same noise level [37]. However, the position of the RPE between these two layers likely influences the signal. In contrast, the standard deviation (SD) method uses the mean and SD of a reference normal database to create a global threshold. Pixels with an intensity lower than one SD below the normal database mean are considered to represent flow deficits [2]. The limitations of this method include the lack of a globally approved and validated normal database and the use of one standard deviation as a threshold, which is debated. Currently, the most commonly used approach is the Phansalkar local thresholding method [1,10,12,26,27,38,39,40,41,42,43,44]. This method sets a circle with a certain “Phansalkar radius” around each pixel, and creates a threshold based off of the intensity mean and standard deviation inside the circle. The limitation of this method is that the Phansalkar radius has to take into consideration the pixel size of the image. Lastly, the fuzzy C-means self-clustering algorithm has recently been proposed, which automatically assigns all pixels of the inner choroidal slab into clusters based on their histogram distribution [45]. Such variety and the lack of a globally accepted and validated method complicates the quantitative study of the choriocapillaris considerably.

## 3. OCTA of the Choriocapillaris in Normal Aging

Analysis of the choriocapillaris in AMD must be considered in the context of the normal aging of the retina. A histopathologic study shows that age is highly correlated with decreasing density of the choriocapillaris, particularly in the macula [46]. These normal age-dependent changes in choriocapillaris flow characteristics can be identified in vivo with OCTA. An early SD-OCTA study demonstrated that the number of flow voids increase with age [47] in a power law distribution. Subsequent SS-OCTA studies similarly demonstrated an increase in the flow deficit percentage within the macula and an increase in the variability of flow deficit measurements with age [43]. These changes may be secondary to underlying systemic vascular conditions that are common in older individuals, such as hypertension, which itself has been shown to increase the number of choriocapillaris flow voids [47], although systemic vascular disease as the cause of age-related OCTA choriocapillaris impairment has not been studied robustly. Interestingly, the central 1 mm circle of the macula shows the greatest increase in percentage of flow deficits with age [10,43]. Age-related localized ischemia in the central macula may contribute to drusen and macular neovascularization (MNV) development, especially considering that soft drusen and MNV are found more commonly in the central macula [48,49,50], although this relationship remains speculative. It is also important to consider that increased choriocapillaris flow voids, as attributed to normal aging, can also be found in early AMD. The OCTA findings associated with normal aging and the OCTA findings that occur during transition to early AMD are not yet well defined.

## 4. OCTA of the Choriocapillaris in Non-Neovascular AMD

Choriocapillaris disease is associated with outer retinal, RPE and Bruch’s membrane disruption in all stages of AMD. Several histopathologic studies have shown that choriocapillaris density decreases with increasing AMD severity [51,52]. It should be recognized, however, that there is some inconsistency in these findings in the literature. The choriocapillaris in advancing AMD can display decreased density [46], increased density [53], or equivocal features [54] according to various reports. This may be in part attributable to an underestimation of vascular cell death with hematoxylin and eosin staining methods compared to the use of endothelial markers like UEA-I lectin [55]. However, various OCTA studies, discussed below, have identified distinct and significant choriocapillaris alterations in the eyes of patients with intermediate and late-stage AMD and have shown that the health of the choriocapillaris may be an important predictive factor. These findings should be considered within the context of the ongoing discussion and limitations of choriocapillaris imaging and quantitative analysis described in Section 2.

### 4.1. Choriocapillaris Impairment in Non-Neovascular Intermediate AMD

Early outer retinal abnormalities in non-neovascular AMD are associated with impairment of the choriocapillaris. Drusen, the hallmark feature of early AMD, is associated with progressive disruption of the RPE, Bruch’s membrane and choriocapillaris [56]. Histopathologic [57] and OCT [58] studies have shown that choriocapillaris loss can be co-localized with some, but not all, drusen. This association has been corroborated by OCTA analysis of intermediate AMD eyes, which has displayed impaired choriocapillaris flow [47], particularly beneath and surrounding drusen [59]. As discussed above, shadow artifact underlying drusen can give the false impression of choriocapillaris flow deficits [29,60], although image compensation strategies can mitigate these quantification limitations. Patients with reticular pseudodrusen show unique choriocapillaris alterations, with lower choroidal thickness and volume, higher choroidal vascular index, and higher choroidal intensity [61]. These early choriocapillaris changes may have functional impacts as well; choriocapillaris flow impairment was found to correlate with reduced scotopic macular sensitivity in eyes with early or intermediate AMD [62]. In these ways, the health of the choriocapillaris on OCTA is a meaningful indicator of the severity of disease in AMD.

The health of the choriocapillaris on OCTA may also have predictive power in determining the advancement of disease. In patients with macular drusen, choriocapillaris flow deficit predicts both the enlargement of the existing drusen and the development of new drusen [63]. Impairment of the choriocapillaris may also indicate progression to more advanced stages of the disease. Choriocapillaris flow deficits are worse in patients with hyperreflective foci, particularly directly under the hyperreflective focus [64]. These hyperreflective foci correlate with progression to late AMD and development of atrophy [65]. More directly, choriocapillaris flow deficit itself can predict progression of disease. Greater inner choroidal flow deficit can be a predictor of progression to incomplete RPE and outer retinal atrophy (iRORA) [66]. Similarly, choriocapillaris flow deficit is greater in intermediate AMD eyes that progress to complete RPE and outer retinal atrophy (cRORA) [41]. Guided by these findings, OCTA of the choriocapillaris may provide useful risk stratification or predictive benefits in the future.

### 4.2. Choriocapillaris Impairment in Geographic Atrophy

**Figure 1:**
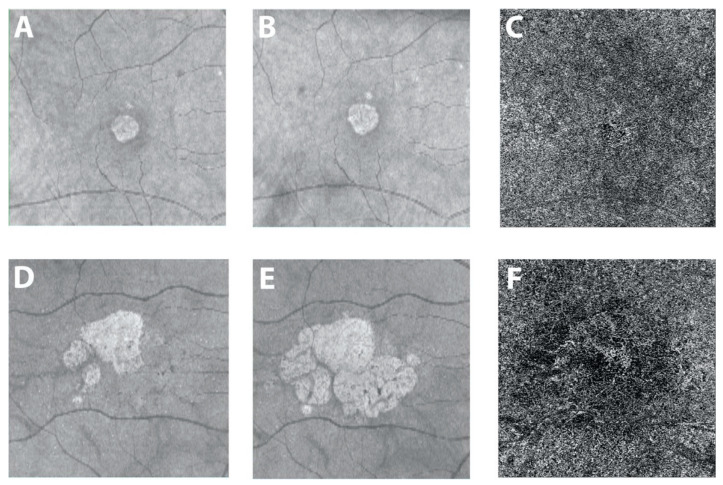
Courtesy of Nassisi et al. 2019: Geographic atrophy and different growth rates: One eye from two patients with geographic atrophy (GA) is shown in the two rows of images. (**A**,**D**) En face structural optical coherence tomography (OCT) images at baseline. (**B**,**E**) En face OCT images acquired one year later. The 2 patients show a very different yearly growth rate (0.07 and 0.73 for the first and second row, respectively) of the atrophic lesins. The corresponding OCT angiogram at the level of the choriocapillaris (**C**,**F**) from the baseline visit for these two patients shows dramatically different flow impairment surrounding the atrophic lesion, with significantly greater flow voids in the case with more rapid progression of atrophy (41.2% versus 53% for (**C**,**F**) respectively) [73].

As geographic atrophy (GA) describes the atrophy of the outer retina, RPE and choriocapillaris, it is not surprising that patients with advanced non-neovascular AMD show choriocapillaris alterations on OCTA. OCTA in fact allows for the demarcation of the area of atrophy as accurately as fundus autofluorescence [67]. Impairment of the choriocapillaris can be identified before complete atrophy sets in. Flow impairment is associated with regions of nascent GA [68], a precursor of drusen-associated GA. As the disease progresses to GA, the choriocapillaris exhibits significant impairment underneath the area of atrophy [39,69,70]. As the choriocapillaris is lost, the middle portions of the choroid regress and the deeper larger choroidal vessels ascend to lie in the inner choroid [5,71,72]. It is important to note that the choriocapillaris is also impaired in the peripheral macula in patients with GA, compared to both normal eyes and eyes with CNV [39,68]. The choriocapillaris in the zone immediately surrounding geographic atrophy shows the greatest impairment on OCTA and predicts the rate of atrophy enlargement (Figure 1) [73,74]. These findings again indicate the critical and possibly predictive role of the choriocapillaris and its impairment in the development and progression of GA.

### 4.3. The Role of OCTA in Clinical Management of Non-Neovascular AMD

While the aforementioned findings shed noteworthy light on the understanding of non-neovascular AMD progression, providers should be cautious when using OCTA of the choriocapillaris to guide clinical management. OCTA choriocapillaris flow deficits cannot yet predict AMD progression on an individual case by case basis and the methodology remains a research tool with potentially great clinical importance for the future evaluation of AMD patients. Evaluation of eyes with intermediate AMD and drusen using OCTA can be difficult and the interpretation of images can be challenging. Projection artifacts from the overlying retinal vessels can create pseudoflow and the illusion of neovascularization in eyes with drusen [11]. However, OCTA can accurately detect non-exudative MNV in eyes that would otherwise be classified as intermediate AMD, a lesion that may be present in as many as 25–30% of eyes with intermediate AMD [75]. This holds clear clinical significance, as non-exudative type 1 MNV is predictive of progression to exudative disease [76]. OCTA of the choriocapillaris may be particularly helpful in cases of non-neovascular age-related macular degeneration with subretinal fluid [77] associated with non-vascularized macular drusen and drusenoid pigment epithelial detachments (PEDs). In these cases, OCTA confirmation of the absence of MNV despite the associated presence of subretinal fluid is critical to avoid unnecessary anti-VEGF injection and suggests an alternate transudative mechanism of fluid leakage such as RPE decompensation and pump failure, rather than exudative neovascularization, that may be important contributory pathways of leakage in both neovascular and non-neovascular AMD.

## 5. OCTA of the Choriocapillaris in Neovascular AMD

### 5.1. OCTA of Macular Neovascularization

An important application of OCTA is the assessment of MNV in neovascular AMD. MNV is associated with a high risk of vision loss and may necessitate the frequent injection of anti-VEGF agents and therefore the identification and classification of MNV is of critical significance. The advent of OCTA has transformed the diagnostic power of the clinician to detect and image MNV and has provided insights into the pathophysiology of neovascular AMD.

When coupled with its corresponding structural OCT, OCTA is a powerful tool for both the diagnosis and classification of MNV. It has higher sensitivity and specificity than fluorescein angiography (FA) or indocyanine green angiography (ICGA) [78,79,80,81] and does not require dye injection. However, OCTA does not provide information about dynamic leakage and may miss low flow components of neovascularization such as polypoidal lesions.

**Figure 2:**
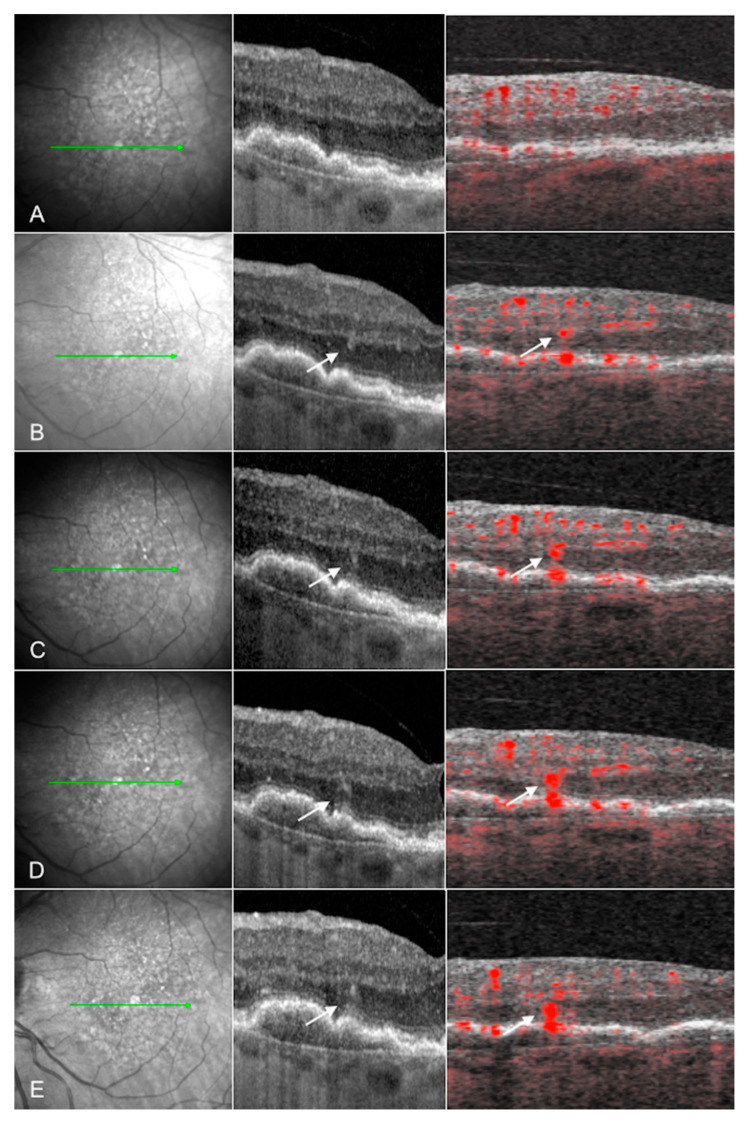
Courtesy of Sacconi et al. 2019: Evolution of type 3 macular neovascularization: Near infrared (NIR) reflectance (left column), structural optical coherence tomography (OCT) B-scan (middle column), and cross-sectional OCT angiography (OCTA) (right column) images of one eye with a type 3 macular neovascularization (MNV) from a patient with age-related macular degeneration (AMD) at (**A**) the first preclinical stage examination and after (**B**) 5 months, (**C**) 8 months, (**D**) 12 months, and (**E**) 15 months. The MNV originates from the deep retinal capillary plexus, evident as a small intraretinal hyperreflective focus on OCT and a punctate flow signal on cross sectional OCTA (white arrows). The lesion progresses downward toward the RPE over time (from (**A**) to (**E**)). These lesions may be driven by choriocapillaris ischemia, as eyes with type 3 MNV have significantly increased choriocapillaris flow deficits with OCTA [95,96].

Beyond identification, OCTA facilitates classification of the neovascularization. MNV can be classified by anatomical position: type 1 MNV is located below the RPE and originates from the choroid, type 2 MNV is located in the sub-retinal space and originates from the choroid, and type 3 MNV is located in the neurosensory retina and originates from the deep retinal capillary plexus and is also known as retinal angiomatous proliferation or RAP [82,83]. Within these classifications, MNV can be sub-classified based on the morphology of the vessels. Mature type 1 MNV is characterized by large thick branching vessels with secondary finer capillary ramification. Various patterns of large vessel branching have been described, including “medusa”, “seafan” and “tangled” morphologies, but these subclassifications have limited clinical value [84]. Hypermature type 1 MNV is characterized by larger vessels without the secondary capillary ramification (“dead tree” pattern) [85,86,87,88,89,90]. The vascular morphology of Type 2 MNV [91,92] is comparable to that of Type 1 MNV, and thus these two MNV types can only truly be differentiated based on their position relative to the RPE. Type 3 MNV originates from the retinal deep capillary plexus (DCP) rather than the choriocapillaris and may begin as a small punctate intraretinal flow signal evident on cross sectional OCTA and can be identified as a small tuft of vessels with en face OCTA. Nascent Type 3 lesions may show progressive downgrowth towards the RPE (Figure 2) [93,94,95]. It is interesting that one study noted increased choriocapillaris nonperfusion compared to fellow non-neovascular eyes, implying that choriocapillaris ischemia may play a critical role in the development of these lesions [96]. Furthermore, this study noted greater choriocapillaris non perfusion in the non-neovascular fellow eyes of patients with type 3 MNV (in the first eye) versus the fellow eyes of patients with Type 1 MNV (in the first eye).

**Figure 3:**
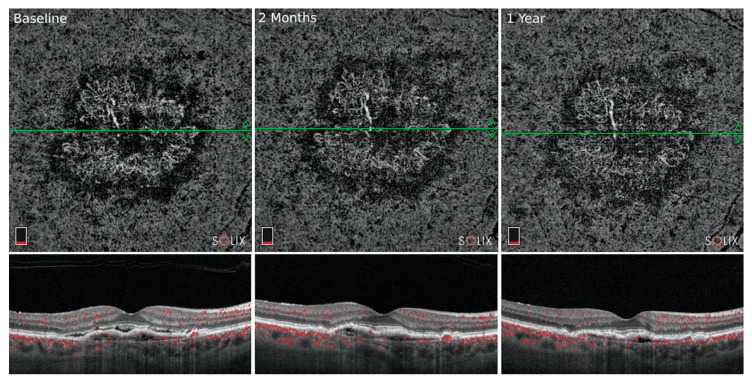
Growth of macular neovascularization after anti-VEGF therapy: En face choriocapillaris OCTA and structural OCT B-scan images of mature type 1 macular neovascularization (MNV). This patient with neovascular age-related macular degeneration (AMD) in the right eye received anti-VEGF injections every 8 to 12 weeks for several years. Note the OCTA characteristics of mature MNV that include larger thick vessels associated with a secondary dense fine capillary ramification. A prominent perilesional halo is also identified with all three lesions. Even with continued anti-VEGF injections, the mature network shows mild growth in total area from the baseline visit (**left**) to the one-year follow-up (**right**) visit. While anti-VEGF treatment reduces fine capillary lesions at the border in the short term, lesions typically grow in overall area after 1 year.

The OCTA morphology of neovascularization can correlate with activity. Fine vessels at the advancing edge of MNV, intralesional fractal dimension, and a dark halo surrounding the lesion may indicate exudative activity, while large, ‘dead tree’ like vessels and a paucity of fine branching capillaries may indicate relative quiescence [78,85,97,98,99,100]. These descriptive features, however, do not have predictive value to guide anti-VEGF therapy, nor is it clear whether they can be graded reliably. After anti-VEGF treatment, MNV can show a rapid decrease in the fine capillary vessels typically at the lesion border, as the capillary fringe is less protected by pericytes than the mature feeding or central trunk vessels that are more anti-VEGF resistant (Figure 3) [88,91,101,102,103]. MNV may also temporarily decrease in size after anti-VEGF therapy, but subsequently increase in size after two weeks [104]. With repeated anti-VEGF injections, often only the mature, “tree trunk-like” pericyte protected vessels remain, after which lesions are referred to as “mature” or “hypermature” [85,105,106]. Chronic anti-VEGF therapy of mature type 1 MNV is associated with progressive growth in the lesion area after 1 year in the majority of cases with various growth patterns [106]. Lesions with extensive vascularity are typically associated with good acuity and lesions with low vascularity are associated with poor acuity.

### 5.2. OCTA of the Choriocapillaris in Neovascular AMD

**Figure 4:**
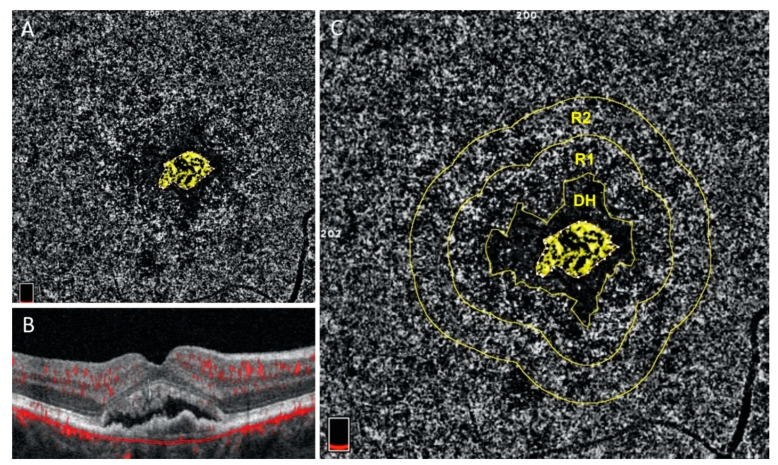
Courtesy of Scharf et al. 2020: Choriocapillaris flow deficits around macular neovascularization: En face choriocapillaris OCTA (**A** and **C**) and structural OCT B-scan (**B**) images of treatment-naïve exudative macular neovascularization (MNV). In the Scharf study, en face choriocapillaris angiograms were analyzed for the percentage of choriocapillaris (CC) flow deficits in two concentric rings, (R1) and (R2), around the peri-lesional dark halo (DH), (image **C**). The ring closer to the MNV (R1) exhibits significantly greater percentage of flow deficits than the more peripheral ring. Both rings exhibit significantly greater flow deficits than the same areas in age-matched normal controls [44].

Beyond the direct study of the neovascular membrane, OCTA studies show that the choriocapillaris is impaired in the environment surrounding the MNV. MNV is commonly encircled by a “dark-halo” on OCTA, an area devoid of flow which may represent a vascular steal phenomenon [107] because of flow diverted through the neovascular membrane [108] or the result of inner choroidal ischemia [44,109]. This dark halo may in fact be a marker of activity of the neovascularization [78,98]. The choriocapillaris immediately surrounding MNV shows higher flow deficits than other areas of the macula [109,110,111]. The association holds true even when analyzing only the choriocapillaris immediately outside of the perilesional dark halo in treatment-naïve eyes (Figure 4) [44]. This suggests that RPE hypoxia caused by choriocapillaris disease may drive VEGF release and MNV development [7,44,112,113], but cannot exclude the possibility that the choriocapillaris impairment is instead secondary to the MNV. It is interesting that choriocapillaris flow deficits may be greater around exudative versus nonexudative MNV and future applications of OCTA may become important to determine which nonexudative NV lesions may be appropriate to treat with anti-VEGF therapy [44]. The choriocapillaris in the peripheral macula remote from the MNV lesion is similar to age-matched normal eyes, which is in contrast to eyes with GA, which show a significant increase in choriocapillaris flow deficit throughout the macula. This has led to the hypothesis that the choriocapillaris in eyes with GA may be so severely impaired, such that it is no longer capable of supporting an MNV response [41,66,74,111]. These concepts will need to be validated in future prospective studies.

## 6. Future Directions

OCTA in vivo study of the choriocapillaris is a remarkable advancement in the field of retinal imaging and AMD. As a relatively novel imaging modality, there is much progress still to be made in both image acquisition and choriocapillaris analysis. Faster scan speeds and advancements in software for motion artifact correction, projection removal, tracking and segmentation have the potential to improve image quality and consistency. Progress in choriocapillaris quantification algorithms may improve the reliability and reproducibility of these measurements and allow for automated analysis which could be clinically applicable. New image analysis algorithms like variable interscan time analysis (VISTA), which provides information on relative blood flow speed [114], continue to expand the capabilities of OCTA. VISTA has already brought creative insights to the literature, showing unique flow speeds in different parts of the MNV complex [115], varying degrees of flow impairment in particular regions of GA [5], and distinguishing between choriocapillaris flow impairment and complete choriocapillaris atrophy [68]. With continued OCTA hardware and software innovation, advancements in clinical applications and pathophysiologic discoveries in AMD are set to follow.

## Data Availability

No new data were created or analyzed in this study. Data sharing is not applicable to this article.
